# Occulus-Guided Rehabilitation Approach in a Patient With Osteoarthritis Knee: A Case Report

**DOI:** 10.7759/cureus.29021

**Published:** 2022-09-11

**Authors:** Shrushti Jachak, Pratik Phansopkar, Neha Chitale

**Affiliations:** 1 Musculoskeletal Physiotherapy, Ravi Nair Physiotherapy College, Datta Meghe Institute of Medical Sciences, Wardha, IND

**Keywords:** case report, physiotherapy, physical rehabilitation, virtual reality, osteoarthritis

## Abstract

Osteoarthritis (OA) is the most frequent musculoskeletal condition affecting the elderly's quality of life. Wear and tear, as well as the progressive deterioration of articular cartilage, are the causes of OA, also known as degenerative joint disease. There are two types of OA: primary and secondary. The condition can be treated both conservatively and surgically. Medication and physical therapy might be used to treat it conservatively. Physical rehabilitation can include advanced management through the use of virtual reality (VR). The other option for treatment is surgical management. We are presenting a case of a 52-year-old female who presented to musculoskeletal physiotherapy out patient department with complaints of left-sided knee pain, stiffness, and difficulty in sitting with crossed legs on the floor. On examination, crepitations were present. Knee range of motion and strength were significantly reduced. Physiotherapy treatment was planned for the same. In physiotherapy rehabilitation, Occulus-guided physiotherapy exercises were given to improve the strength and range of motion of the joint.

## Introduction

Osteoarthritis (OA) is the most frequent musculoskeletal issue in the elderly. OA is a significant medical condition that affects people all over the world [1]. It is induced by the degradation of the articular cartilage of the joints and is so usually referred to as degenerative joint disease. Although the cause of OA is unknown, age, genetics, obesity, menopause, hypertension, and diabetes mellitus have all been identified as key risk factors [2]. Its prevalence, which is currently around 3.5% of the world's total population and around 35% in population over the age of 60, is increasing [3]. According to the World Health Organization's Global Burden of Disease Survey, knee OA is anticipated to become the eighth most common cause of disability in men and the fourth most common cause of impairment in women [4]. OA clinical signs include knee weakness and swelling, knee pain that gradually worsens with exercise, and discomfort after long periods of sitting or sleeping. The severity of the ailment determines how the condition can be managed. Conservative therapy and surgical treatments can be used to alleviate the condition. Medication and physical therapy, which may involve virtual reality (VR), can be used to manage it conservatively [5]. VR can potentially aid healing by decreasing pain, diverting from pain worries, and lowering stress levels [6]. Physical therapy plays an important role to maintain the quality of life of patients with OA to knee. Including VR as a treatment approach makes the treatment more beneficial to the patients.

## Case presentation

A 52-year-old female presented to the musculoskeletal physiotherapy outpatient department with complaints of left-sided knee discomfort, stiffness, and difficulties sitting with her legs crossed on the floor. After a long period of sitting, the patient complains of pain. She was extensively evaluated. On evaluation Clark test was positive, pain was in patellar region with patella slightly shifted medially and following the physiotherapeutic evaluation, the patient was advised for the X-ray done. An X-ray revealed evidence of grade II knee OA. The diagnosis was verified, and physical therapy was initiated.

Clinical findings

**Figure 1:**
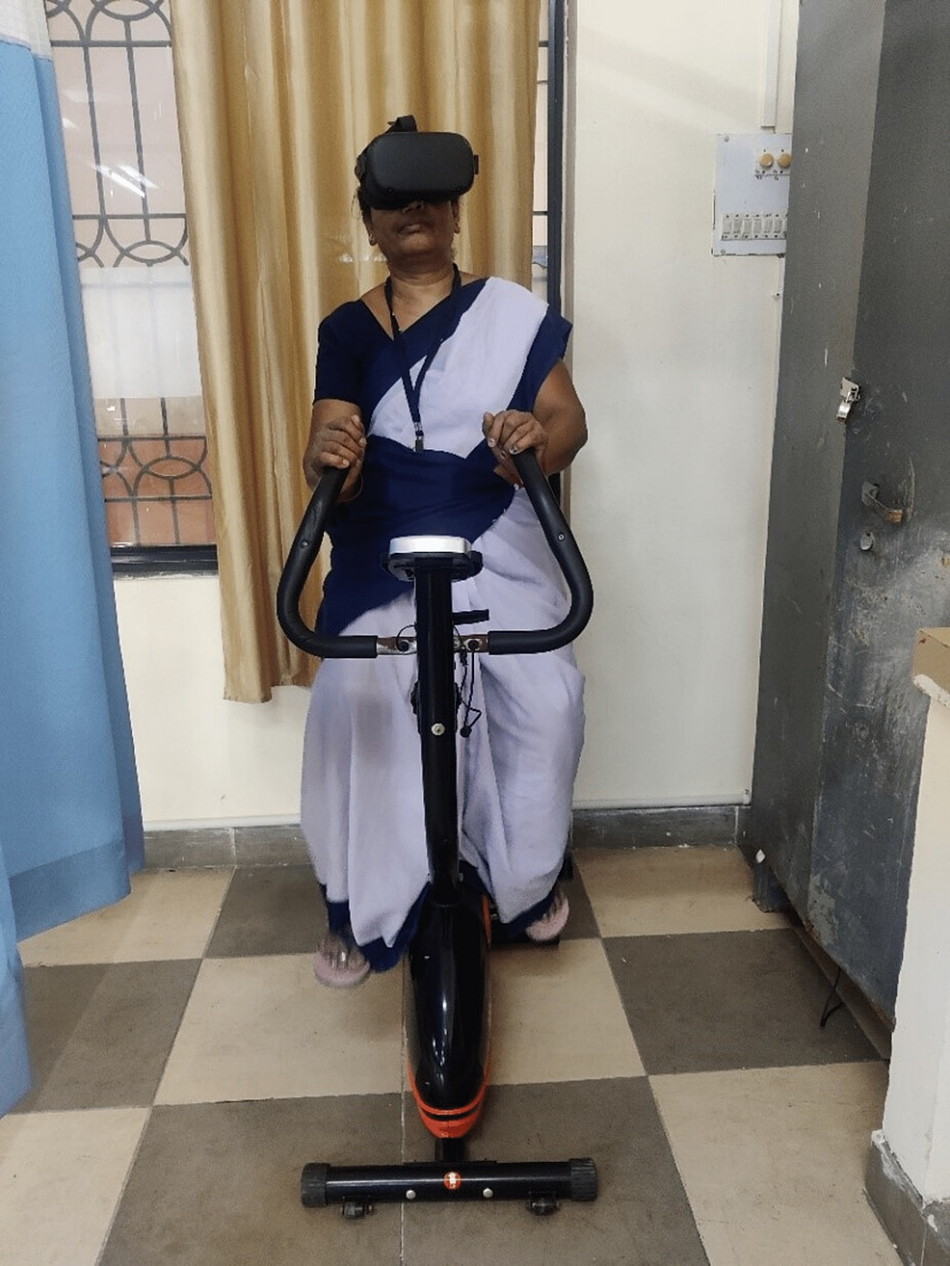
Patient performing static cycle with occulus.

The patient offered informed oral consent. The patient scored the pain as 8/10 on the Numerical Pain Rating Scale (NPRS), with a gradual onset. The pain was in the medial aspect of the left knee and was dull aching in nature. Pain was exacerbated by activities, and the relieving factors were rest and medicine. A patient complained of stiffness in the morning. The swelling was visible upon inspection. On palpation, there was grade 3 tenderness. Crepitations were evident, and an empty end feel was present. On examination, muscle strength was reduced, which can be seen in Table 1. And there was decreased range of motion, in hypomobile joints, as shown in Table 2. The patient was advised for the X-ray, and the investigation confirmed the grade 2 OA (Figure 1). WOMAC score was 54. Special tests namely Patellar tab test and Grind test were positive.

Physiotherapy management 

The patient's management goals include reducing pain intensity, enhancing joint range of motion, and making the patient as independent as practical. The patient was started on a four-week physiotherapy rehabilitation plan, which included the following:

Week 1

The first week of treatment included ultrasound therapy (3 MHz frequency and 0.25-1 W/cm^2^ intensity). Isometric quadriceps and hamstring exercises were initiated (10 repetitions x 1 set). Straight leg lifts with a three-second hold (10 repetitions x 1 set). The patient was instructed to walk for at least 20 min/day. The patient was treated with VR using occulus, and the patient was given a 10-min session with the ExrcsBik game (Figure 1). Retro walking for 10 min twice a day was recommended (Figure 1).

Week 2

Maitland mobilization was added to the existing regimen in the second week of treatment to reduce discomfort and enhance functional range of motion.

Week 3

The dynamic quadriceps exercises were initiated (10 repetitions x 1 set). By this time, knee strengthening exercises with a weight cuff of 1 kg had begun. The patient was treated with occulus. The patient was given a 20-min session with the ExrcsBik game. Maitland mobilization and ultrasound were both continued.

Week 4

Knee strengthening exercises using therapeutic bands, tubes, and a weight cuff were initiated. The rest of the protocol remained unchanged. The patient was encouraged to walk for 30 min every day.

Follow-up and outcome

Follow-up was taken weekly, once for one month. There was a significant reduction in pain and improvement in range of motion. The patient was able to perform her everyday activities independently (Tables 1-2).

**Table 1:** Range of motion (pre-treatment and post-treatment).

Joint	Movement	Right pre	Right post	Left pre	Left post
Knee	Flexion	0-120^0^	0-125^0^	10-90^0^	0-120^0^
	Extension	120-0^0^	125-0^0^	90-10^0^	120-0^0^

**Table 2:** Manual muscle testing (pre-treatment and post-treatment).

Joint	Muscles	Grade pre	Grade post
Knee	Flexor	3	5
	Extensor	2	4
Hip	Flexor	4	5
	Extensor	3	5
	Adductors	3	5
	Abductors	3	4

## Discussion

Knee OA is a frequent condition. As a result, many studies that included physical therapy management in addition to conservative medical management have been done. Many trials used either VR [6] or traditional physical treatment, but this is the first time we have seen a combination of the two. Loyola-Sánchez et al. studied how effectively ultrasound therapy reduced pain and improved function in persons with knee OA, as well as patient understanding of disease severity and cartilage repair. The findings showed a significant improvement in pain and function in patients with knee OA [7].Using technology to deliver fitness services continues to be advantageous, according to a comprehensive analysis conducted by Chen et al. The sorts of technology and software aspects connected to positive health outcomes have been identified, along with recommendations for additional research and deployment of those systems [8].

Pozsgai et al. observed that short-term end-range Maitland mobilisation can diminish peripheral and central sensitization, resulting in a greater pain threshold in knee OA patients [9]. In addition, because other pain-related therapies have proven to be successful, end-range Maitland mobilization can improve the functional status of patients with knee OA very quickly. As a consequence, they suggest that Maitland mobilization be utilized not just during longer periods of rehabilitation, but also in outpatient settings to enable patients with knee OA swiftly improve their functional status [9]. Retro walking, according to Wadhwa, is beneficial in lowering pain and disability in knee OA patients and can be used as an alternative to standard therapy [10].

Exergaming (exercise and gaming) can help improve balance in a variety of health issues, but there is no data on its efficacy in people with knee OA. Exergaming is the future of rehabilitation. It has a wide variety of application [11]. Despite the fact that the exergame intervention procedures and outcome measures for measuring intervention efficacy differed, the data amassed demonstrated that exergame therapies enhanced physical or cognitive abilities in the elderly [12]. The exergaming and VR engross the patients and hence helps in achieving the desired benefits in the patient. VR can help in cognitive and motor rehabilitation [13]. The use of specialist VR and gaming VR can be beneficial for upper extremity therapy, but not for hand dexterity and gait in all disorders studied. Neurological sufferers can benefit from specialized VR [14]. In terms of shoulder impingement syndrome and persistent neck discomfort, the evidence of VR's usefulness is encouraging [5]. In cases of rheumatoid arthritis, OA of the knee, ankle instability, and post-anterior cruciate reconstruction, VR and workouts show comparable results. There is no or conflicting evidence that VR is more useful than exercise for conditions including fibromyalgia, back pain, and knee arthroplasty. We have seen the positive effects of VR training in this study in reducing the symptoms in the patients, and that was seen in the outcome measures used in this study [15]. We noticed muscle strengthening had increased the stability of the joint and helped patient regain functional activities. Exercise programs that adhere to the American College of Sports Medicine's standards for strength training produce better results in terms of knee strength but not in terms of pain or impairment [16].

## Conclusions

The patient had complaints of pain and stiffness for which physiotherapy in the form of VR was given. The patient was provided a comprehensive strategy for increasing strength and range of motion. As a result of the therapy, the patient's condition considerably improved. The patient was able to carry out her daily tasks on her own. There were no doubts in the patient's mind. In conclusion, this case study demonstrates that a patient with knee OA recovered entirely with the help of occulus-guided physical therapy

## References

[1] Kobayashi S, Pappas E, Fransen M, Refshauge K, Simic M (2016). The prevalence of patellofemoral osteoarthritis: a systematic review and meta-analysis. Osteoarthritis Cartilage.

[2] Cimmino MA, Cutolo M (1990). Plasma glucose concentration in symptomatic osteoarthritis: a clinical and epidemiological survey. Clin Exp Rheumatol.

[3] Goel S, Kamath SU, Annappa R (2021). Cross-sectional assessment of cardiovascular risk factors in patients with knee osteoarthritis. F1000Res.

[4] Lopez AD, Murray CC (1998). The global burden of disease, 1990-2020. Nat Med.

[5] Lin HT, Li YI, Hu WP, Huang CC, Du YC (2019). A scoping review of the efficacy of virtual reality and exergaming on patients of musculoskeletal system disorder. J Clin Med.

[6] Hoffman HG, Patterson DR, Carrougher GJ (2000). Use of virtual reality for adjunctive treatment of adult burn pain during physical therapy: a controlled study. Clin J Pain.

[7] Loyola-Sánchez A, Richardson J, MacIntyre NJ (2010). Efficacy of ultrasound therapy for the management of knee osteoarthritis: a systematic review with meta-analysis. Osteoarthritis Cartilage.

[8] Chen T, Or CK, Chen J (2021). Effects of technology-supported exercise programs on the knee pain, physical function, and quality of life of individuals with knee osteoarthritis and/or chronic knee pain: A systematic review and meta-analysis of randomized controlled trials. J Am Med Inform Assoc.

[9] Pozsgai M, Péter AI, Farkas N, Than P, Than P, Nusser N (2020). The immediate effect of end-range maitland mobilization on pain pressure threshold in patients with knee osteoarthritis. Osteoarthritis Cartilage.

[10] Wadhwa DN (2016). Effects of retrowalking on osteoarthritis of knee in geriatric population. IOSR J Sports Phys Educ.

[11] Manlapaz DG, Sole G, Jayakaran P, Chapple CM (2021). Exergaming to improve balance and decrease the risk of falling in adults with knee osteoarthritis: a mixed-methods feasibility study. Physiother Theory Pract.

[12] Choi SD, Guo L, Kang D, Xiong S (2017). Exergame technology and interactive interventions for elderly fall prevention: a systematic literature review. Appl Ergon.

[13] Tieri G, Morone G, Paolucci S, Iosa M (2018). Virtual reality in cognitive and motor rehabilitation: facts, fiction and fallacies. Expert Rev Med Devices.

[14] Rutkowski S, Kiper P, Cacciante L, Cieślik B, Mazurek J, Turolla A, Szczepańska-Gieracha J (2020). Use of virtual reality-based training in different fields of rehabilitation: a systematic review and meta-analysis. J Rehabil Med.

[15] Gumaa M, Rehan Youssef A (2019). Is virtual reality effective in orthopedic rehabilitation? A systematic review and meta-analysis. Phys Ther.

[16] Bartholdy C, Juhl C, Christensen R, Lund H, Zhang W, Henriksen M (2017). The role of muscle strengthening in exercise therapy for knee osteoarthritis: a systematic review and meta-regression analysis of randomized trials. Semin Arthritis Rheum.

